# Evolving Trauma Demands: Trends in Orthopaedic Case Volume and Major Trauma Centre Service Adaptations, 2016–2024

**DOI:** 10.7759/cureus.97364

**Published:** 2025-11-20

**Authors:** Archie Allen, Luke Crocker

**Affiliations:** 1 Trauma and Orthopaedics, North Bristol National Health Service Trust, Bristol, GBR; 2 Trauma and Orthopaedics, University Hospitals Bristol and Weston National Health Service Foundation Trust, Bristol, GBR

**Keywords:** fractures, major trauma centre, rising trauma volume, service planning, theatre utilisation, trauma epidemiology

## Abstract

Background

Healthcare services in the UK are becoming increasingly stretched, attempting to match the demands of an ageing and growing population, with Trauma and Orthopaedics being no exception. New national targets to reduce waiting times have resulted in additional operating capacity for elective services; however, trauma services have not seen an equivalent expansion.

Aim

This retrospective operational audit aims to quantify changes in trauma workload in a Major Trauma Centre between 2016 and 2024 and assess how the service has adapted to accommodate these changes, to aid service planning.

Methods

A retrospective operational audit was conducted in a UK Major Trauma Centre using archived daily trauma meeting lists (n=237) and operation records (n=1376), from June and November of 2016, 2019, 2021 and 2024. Linear regression and a sign test were used to test for significance. From this data, we extrapolated ways in which the service has adapted to meet the changes in demand.

Results

Whilst the number of trauma operations did not change considerably between 2016 (n=344) and 2024 (n=359, a 4.36% increase), there was a substantial increase in many time-sensitive operations including a 47% increase (from n=34 to n=50) in open fracture operations, a 70% increase (from n=20 to n=34) in femoral fracture operations, and an 11% increase (from n=90 to n=100) in hip fracture operations. Upper limb fracture operations increased by 46% (from n=41 to n=60); however, ankle fracture operations only saw a 2.9% increase (from n=35 to n=36). Limited data meant individual operation types did not show significant increases; however, the overall upward trend of all fracture types studied was significant (p=0.016). These changes were accommodated for by reallocating several procedures to elective lists, including arthroscopic repairs, prosthetic joint infections, and removal of metalwork.

In 2016, nine trauma operations were conducted in elective or additional theatre lists. This rose to 88 in 2021 when spare elective lists were available following the COVID-19 pandemic, before falling to 45 in 2024 after full elective services returned.

The mean average number of patients discussed in daily trauma meetings in June 2024 increased by 19% (from 28.7±6.6 in 2016 to 34.1±6.9 in 2024) and 25% in November (from 23.9±3.3 in 2016 to 29.8±7.8). The average number of operations listed for the day increased by 22% (from 6.01±2.55 in 2016 to 7.34±2.75) in 2024.

Conclusions

The substantial increase in volume of time-sensitive trauma patients has forced the service to adapt through changes in theatre utilisation. To create sufficient space in trauma theatres, many procedures have migrated to elective lists. Spare theatre time on elective lists is regularly used to complete trauma cases when the trauma workload exceeds capacity. Ad hoc additional theatres are more regularly opened for trauma when demand exceeds capacity.

Patient care is already being impacted through increased delays to treatment. Trauma volume is predicted to continue rising with the growing ageing population, and without additional trauma lists or semi-elective ambulatory lists, trauma services will soon become overwhelmed, undermining the ability to provide safe and timely care for patients.

## Introduction

Healthcare services in the United Kingdom are experiencing mounting pressure from an ageing and expanding population, and Trauma and Orthopaedics is no exception. The UK population continues to grow and the number of adults aged 65 and over is ever increasing [1], but disease-free life expectancy is declining [2]. Patients are increasingly spending a greater proportion of their later years burdened by frailty and multiple co-morbidities [3]. In orthopaedics, this translates into a growing burden of fragility and periprosthetic fractures [4,5]. Alongside fragility fractures, the growing working-age population continues to present with high-energy injuries, creating a dual demand on trauma services that is both rising and increasingly complex.

Elective waiting lists currently attract high political and media attention, so much so that reducing hospital waiting lists is one of the incumbent government’s major policies [6]. Within surgical specialties, the prioritisation of elective procedures has resulted in £1.2 billion of funding from the Targeted Investment Fund to aid the establishment of elective surgical hubs [7]. However, despite comparable increases in demand, trauma services have not seen equivalent prioritisation for investment and service expansion, resulting in increased delays to treatment [8].

The clinical consequences of these delays are well recognised. The National Hip Fracture Database reports that patients with hip fractures operated on within 36 hours of admission have significantly lower mortality and complication rates, yet service pressures meant that in 2024 as many as 42% of patients breach this target compared with 32% prior to the COVID-19 pandemic [9]. For open fractures and long-bone injuries, delays to debridement and fixation increase the risk of infection, non-union, and prolonged disability [10,11]. At a systemic level, inadequate trauma capacity contributes to extended hospital length of stay, high bed occupancy, and delays to onward rehabilitation, exacerbating bottlenecks across acute services [12].

The COVID-19 pandemic temporarily altered this dynamic. National lockdowns were associated with a significant reduction in trauma workload [13]. Additionally, after the lifting of social restrictions, elective services were slow to get back to full capacity, and some trauma services were able to make use of empty elective lists for trauma overflow. As elective surgery resumed, however, pressure shifted back to trauma lists, which now face rising demand with limited additional capacity.

Previous research has demonstrated the number of admissions for fractures in the UK has increased, from 261,556 finished consultant episodes in 2004-2005 to 324,770 in 2013-2014 [14]. Whilst the National Major Trauma Registry continues to collect data on trauma admissions nationwide, there is a relatively little recently published trauma epidemiological data in the UK for the last decade. Similarly, there is limited publicly available research that specifically seeks to aid trauma service planning.

This operational audit aims to quantify changes in orthopaedic trauma workload between 2016 and 2024 in a UK Major Trauma Centre (MTC) and describe how the service has adapted its practice to accommodate these changes. In doing so, it seeks to aid clinicians and policy-makers when planning trauma services. We hypothesised that time-critical trauma operations have risen, and the service has had to adapt to accommodate the increase.

## Materials and methods

A retrospective operational audit was performed of orthopaedic trauma case volume and theatre utilisation in June and November of the years 2016, 2019, 2021 and 2024, at our regional UK Major Trauma Centre (MTC). These months were selected to provide one summer and one winter month, and to avoid national holiday periods with reduced staffing and theatre capacity.

The service

This hospital is the regional MTC for ages 16 and over. It houses the region’s orthoplastic, complex trauma and prosthetic joint infection (PJI) services, alongside other surgical specialties. Today’s orthopaedic service excludes any spinal pathology, hand fractures from the distal carpal row onwards, soft tissue infections (unless involving a joint) and diabetic foot pathology, all of which are managed by separate specialties.

The orthopaedic trauma service has two allocated theatres, providing a total of 32 weekly theatre sessions (where one session is defined as a morning, an afternoon, or an evening list). On Mondays and Fridays, this list is reserved for joint orthoplastic cases. There is a further orthoplastic list on a Wednesday in a designated plastics theatre. Elective orthopaedic lists run every Monday-Friday in separate theatres.

With the recent upgrade of the region’s PJI service and revised referral guidelines, most PJIs are now managed on elective lists under PJI specialists. Patients admitted via the emergency department (ED) with suspected PJIs are initially included within the orthopaedic trauma take, before care is taken over by PJI specialists for further management. Currently, only unwell patients with PJIs requiring early intervention are managed in trauma theatres.

Data collection

All data were collected into a Microsoft Excel (Microsoft Inc., Redmond, WA) spreadsheet.

Operation and theatre utilisation data were manually collected by authors A.A. and L.C. from the hospital’s theatre management software, Bluespier (Lanas (previously Clanwilliam), Dublin, Ireland). Inclusion criteria comprised the following: patient aged 16 and above; case discussed in morning trauma meeting; operation performed in any theatre at the Major Trauma Centre. For multiply injured patients requiring more than one procedure, individual procedures were counted separately. Exclusion criteria included any patient aged 15 and below, and any operation where there was no orthopaedic surgeon involved.

In total, data on 1384 operations were collected that satisfied the inclusion criteria. Of this, there were no patients aged 15 or below, and eight operations where no orthopaedic surgeon was involved were excluded, leaving 1376 operations included for analysis.

For all cases that met the inclusion and exclusion criteria, data was collected on the date of operation, diagnosis, type of operation, and the theatre where the operation was performed (be it trauma theatre, elective theatre, emergency theatre or ad hoc theatre).

To identify trends in the volume of cases discussed in the daily trauma meeting, archived meeting lists from every day of the eight audited months between 2016 and 2024 were included. Three lists were missing from the archives - one from each of June 2016, June 2019 and June 2024, leaving 237 lists for data collection and analysis. From these lists, data were collected on the total number of patients discussed each day and the total numbers of patients listed each day for an operation. These total counts were standardised across the audited period: spinal patients (discussed in trauma meetings in 2016 but not thereafter) and pending aspirate cultures (introduced to the trauma list in 2018) were excluded from the count so comparisons could be drawn between audited years.

Data processing

Following data collection, operations were categorised by type into open fractures, pelvis and acetabular fractures, hip fractures, femoral fractures, tibial fractures, ankle fractures, upper limb fractures and ‘other’. All open fractures, regardless of anatomical location, were grouped under ‘open fractures’ only, with any staged procedures counted individually. ‘Hip fractures’ included intracapsular and extracapsular neck of femur fractures, and subtrochanteric fragility fractures. ‘Femoral fractures’ included high-energy subtrochanteric fractures, and all mid-shaft, distal femoral and peri-prosthetic femoral fractures. ‘Tibial fractures’ included all tibial plateau, tibial shaft and tibial pilon fractures. ‘Ankle fractures’ included all unimalleolar, bimalleolar and trimalleolar ankle fractures. ‘Upper limb fractures’ included fractures of the clavicle, scapula, humerus, radius, ulna and the proximal carpal row. All remaining operations were labelled ‘other’, which included septic joints, dislocations, tendon repairs, drainage of abscesses and rarer fracture types, among others. Total counts for each category were calculated for each audited month and year, irrespective of whether on a trauma or elective list.

Operation data from the ‘other’ category were reviewed by senior faculty, who identified multiple procedures that were previously performed regularly in trauma theatres that are now routinely performed in semi-elective trauma lists or elective lists. These procedures were subsequently categorised as ‘elective trauma’ and include arthroscopic repairs, operative treatment of non-unions, removal of metalwork, treatment of loose bodies and treatment of avascular necrosis. The total counts of these ‘elective trauma’ procedures were calculated for each audited month and year.

The total number of PJI operations performed on trauma lists and the total number of trauma cases performed outside of dedicated trauma theatres were counted from the raw data for each audited month.

Statistical analysis

All statistical calculations were performed with Excel’s inbuilt Analysis ToolPak.

For assessing trends in operation type, data from each audited year were first aggregated, and total counts were used to calculate percentage changes between 2016 and 2024. Linear regression analysis was performed with data from all audited years, plotting the number of each operation category against time (year), with corresponding p-values reported. A sign test was used to evaluate whether the total number of positive trends amongst all operation categories was statistically significant. Statistical significance was defined as p<0.05.

For analysis of ‘elective operations’, PJIs and trauma operations performed in elective lists or ad-hoc trauma lists, direct comparisons were made from the raw data due to the relatively small sample sizes.

Data analysis for the number of patients discussed daily and the number of patients listed for an operation daily involved calculating the means and standard deviations for each of the audited months, which were directly compared.

Graphs and tables were produced in Microsoft Excel for visual comparison of the data. Line charts were used to demonstrate trends in operation categories over time, with linear trendlines calculated using Microsoft Excel’s inbuilt functions. A bar chart was used to visually demonstrate the relative change in numbers of trauma operations performed outside of trauma theatre lists. Tables were used to display the number of operations by type, percentage changes, and the results from linear regression analysis.

## Results

Trends in trauma volume

**Figure 1:**
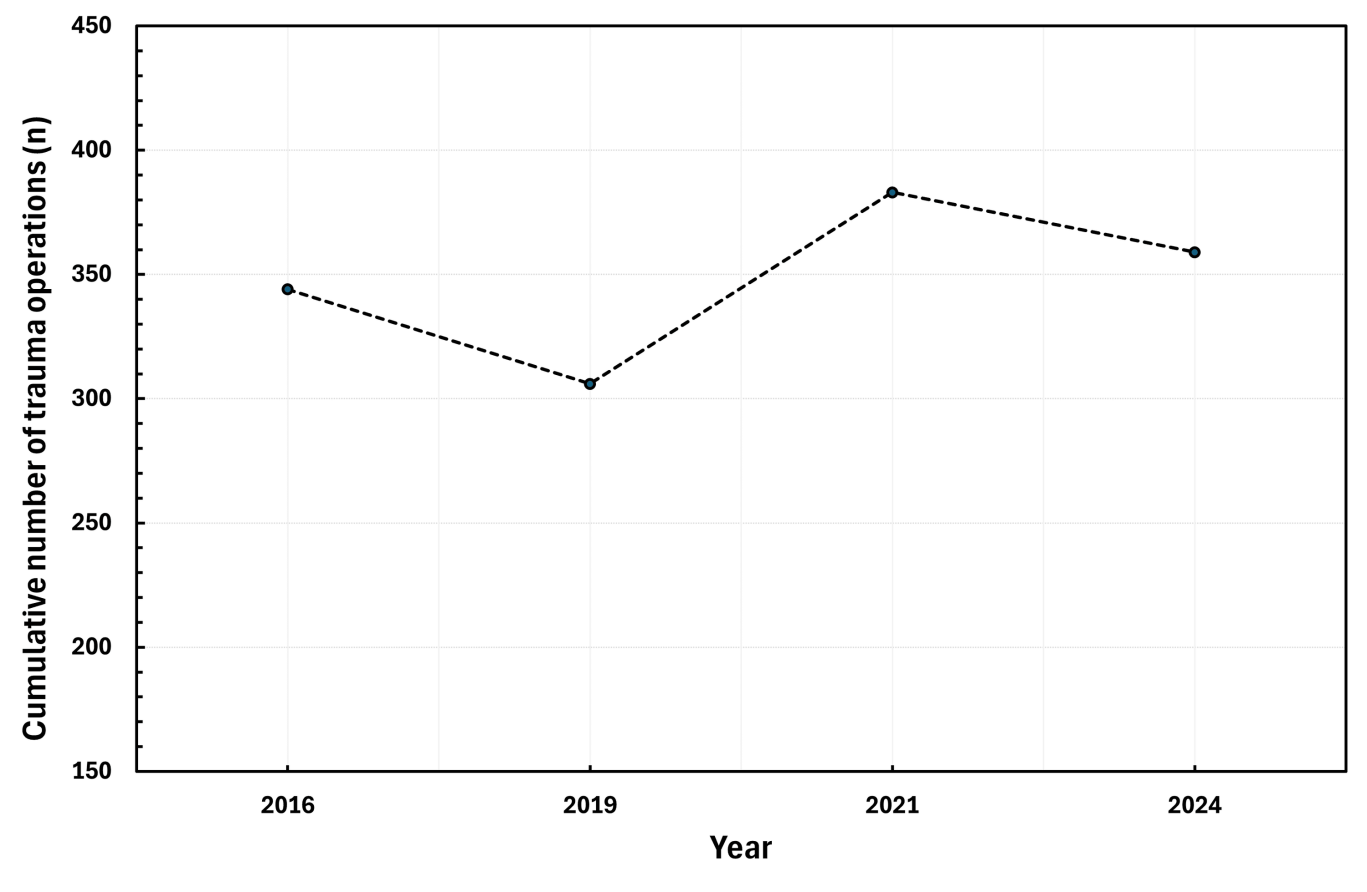
Cumulative number of completed orthopaedic trauma operations by year Line graph demonstrating the cumulative number (n) of orthopaedic trauma operations by year has not changed substantially between 2016 and 2024.

**Figure 2:**
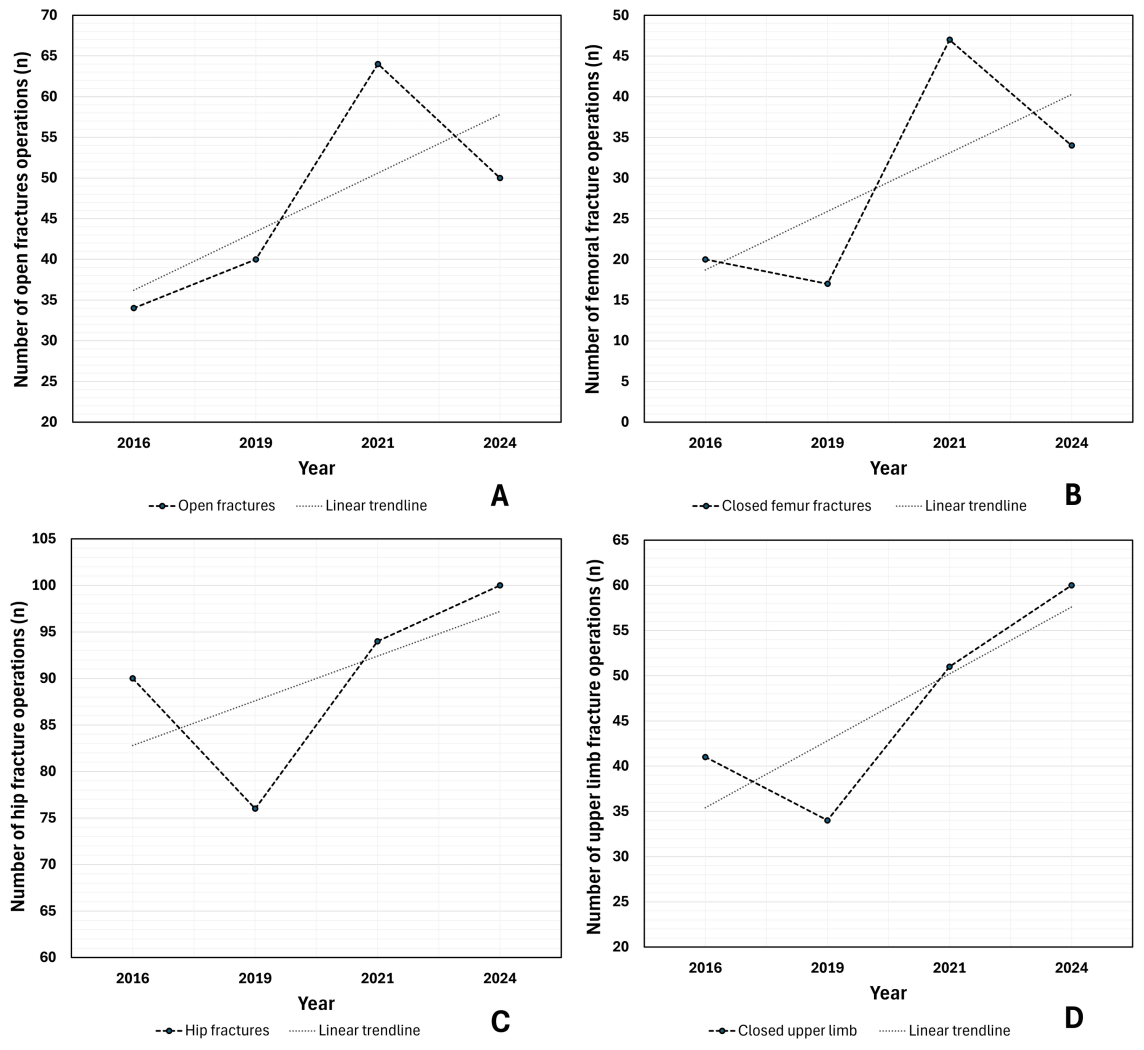
Number of open fracture, femoral fracture, hip fracture and upper limb fracture operations performed by year Line graphs with linear trendlines demonstrating upward trends in numerous operation types, with data aggregated from June and November of each year; A: number (n) of open fracture operations by year; B: number (n) of femoral fracture operations by year; C: number (n) of hip fracture operations by year; D: number (n) of upper limb fracture operations by year

The total number of trauma operations performed has fluctuated but has not increased considerably between 2016 and 2024 (4.4% increase, from n=344 to n=359) (Figure 1, Table 1). Despite this, there has been a substantial increase in many time-sensitive trauma operations. Between 2016 and 2024, open fracture operations have increased by 47% (n=34 to n=50) (Figure 2A), closed femoral fractures by 70% (n=20 to n=34) (Figure 2B), and hip fractures by 11% (n=90 to n=100) (Figure 2C). Additionally, upper limb fracture operations increased by 46% (n=41 to n=60) (Figure 2D). Ankle fractures however have increased by only 2.9% (n=35 to n=36) (Table 1).

**Table 1:** Number of operations in each year by operation type Data on the number of each operation type in each year studied (data from June and November aggregated). Data for each year is presented as number (n), with percentage change between 2016 and 2024 in the final column.

	2016 (n)	2019 (n)	2021 (n)	2024 (n)	Percentage change between 2016 and 2024 (%)
Open fractures	34	40	64	50	+47.1
Hip fractures	90	76	94	100	+11.1
Femoral fractures	20	17	47	34	+70.0
Upper limb fractures	41	34	51	60	+46.3
Pelvis and acetabular fractures	14	16	28	14	0.0
Tibial fractures	15	19	23	17	+13.3
Ankle fractures	35	25	47	36	+2.9
Other	95	79	29	48	-49.5
Total number of operations	344	306	383	359	+4.4

The data for pelvis and acetabular and tibial fractures must be interpreted with caution given the small sample sizes. The volume of pelvis and acetabular fracture operations fluctuated during the study period, increasing from 14 in 2016 to 28 in 2021, before falling to 14 again in 2024 (Table 1). Tibial fracture operations increased by 13.3%, from 15 in 2016 to 17 in 2024 (Table 1).

Linear regression analysis was used to assess for significance of trends in each operation type (Table 2). Whilst there was not enough data to demonstrate a statistically significant increase in any individual operation category, a sign test demonstrated that there has been a significant overall upward trend in trauma operations given seven out of seven analysed operation types had positive slopes on linear regression (p=0.016).

**Table 2:** Linear regressional analysis by operation type Linear regressional analysis of trends in trauma volume from 2016 to 2024 with the audited months substituted for units of time. As demonstrated there were no statistically significant increases when we isolated by operation type. However, a sign test revealed there to be a significant overall upward trend in trauma (p=0.016), given all seven operation types had positive slopes.

Operation type	Slope (operations/unit time)	p-value
Open fractures	1.36	0.28
Hip fractures	1.43	0.37
Pelvis + acetabular fractures	0.12	0.86
Femoral fractures	0.87	0.23
Ankle fractures	0.61	0.4
Tibial fractures	0.07	0.9
Upper limb fractures	1.57	0.098

**Figure 3:**
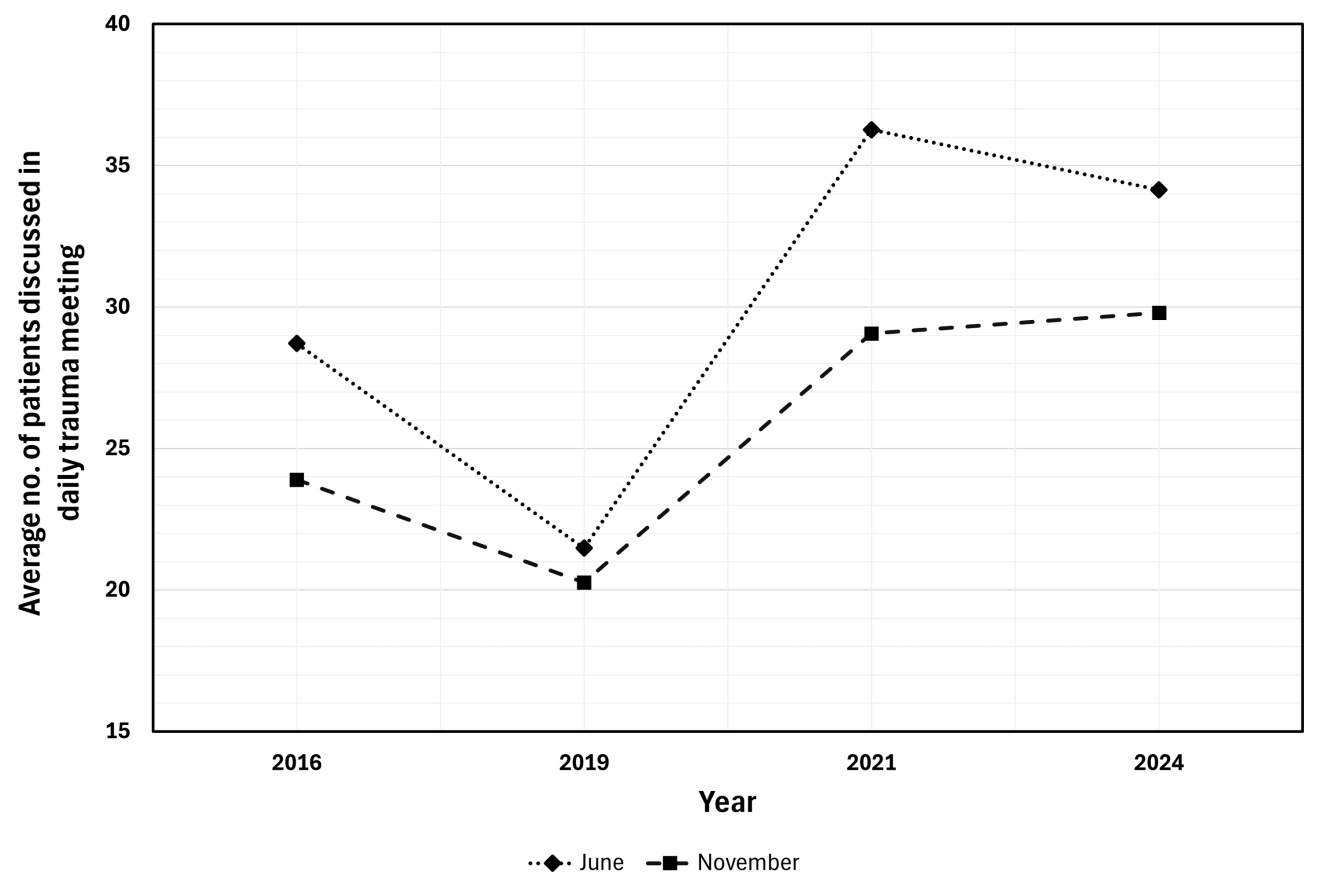
Average number of patients discussed in the daily trauma meeting by month and year Line graph demonstrating the increasing average number of patients discussed in the daily trauma meeting between 2016 and 2024.

As a separate indicator of trends in trauma, we looked at trends the number of patients discussed in the morning meeting and how many patients were listed daily for an operation. The average number of patients on the daily trauma list in June increased by 19% between 2016 and 2024, from 28.7±6.6 to 34.1±6.9, and in November increased by 25% from 23.9±3.3 to 29.80±7.8 (Figure 3). After accounting for 'elective trauma' cases, there was a 22% increase (from 6.01±2.55 in 2016 to 7.34±2.75 in 2024) in the average number of patients listed for an operation that day (irrespective of if completed or not).

Service adaptations to trauma trends

**Figure 4:**
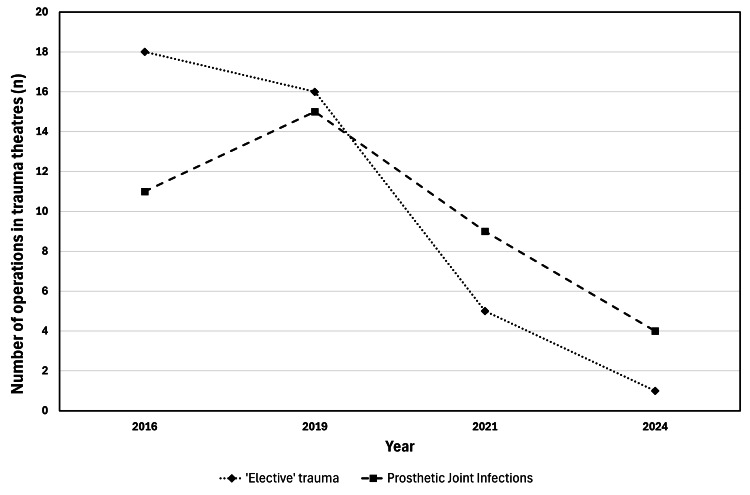
Number of 'elective trauma' and prosthetic joint infection operations being performed in trauma theatres by year 'Elective trauma' refers to operation types that were previously regularly conducted in trauma theatres, which, following recent changes in service delivery, are now routinely performed on elective operating lists. Data represented as n.

The types of operations performed in trauma theatres have changed over the study period as the service has adapted to the changing volume of trauma. Following the pandemic, ‘elective trauma’ including arthroscopic repairs, operative treatment of non-unions, removal of metalwork, treatment of loose bodies and treatment of avascular necrosis were increasingly reassigned from trauma lists to elective lists. There were 18 'elective trauma' operations in 2016 and 16 in 2019, falling to five in 2021 and one in 2024 (Figure 4). Similarly with the restructuring of the regional PJI service, there has been a fall in PJI cases being performed in trauma theatres, from 11 in 2016 and 15 in 2019, to four in 2024 (Figure 4).

**Figure 5:**
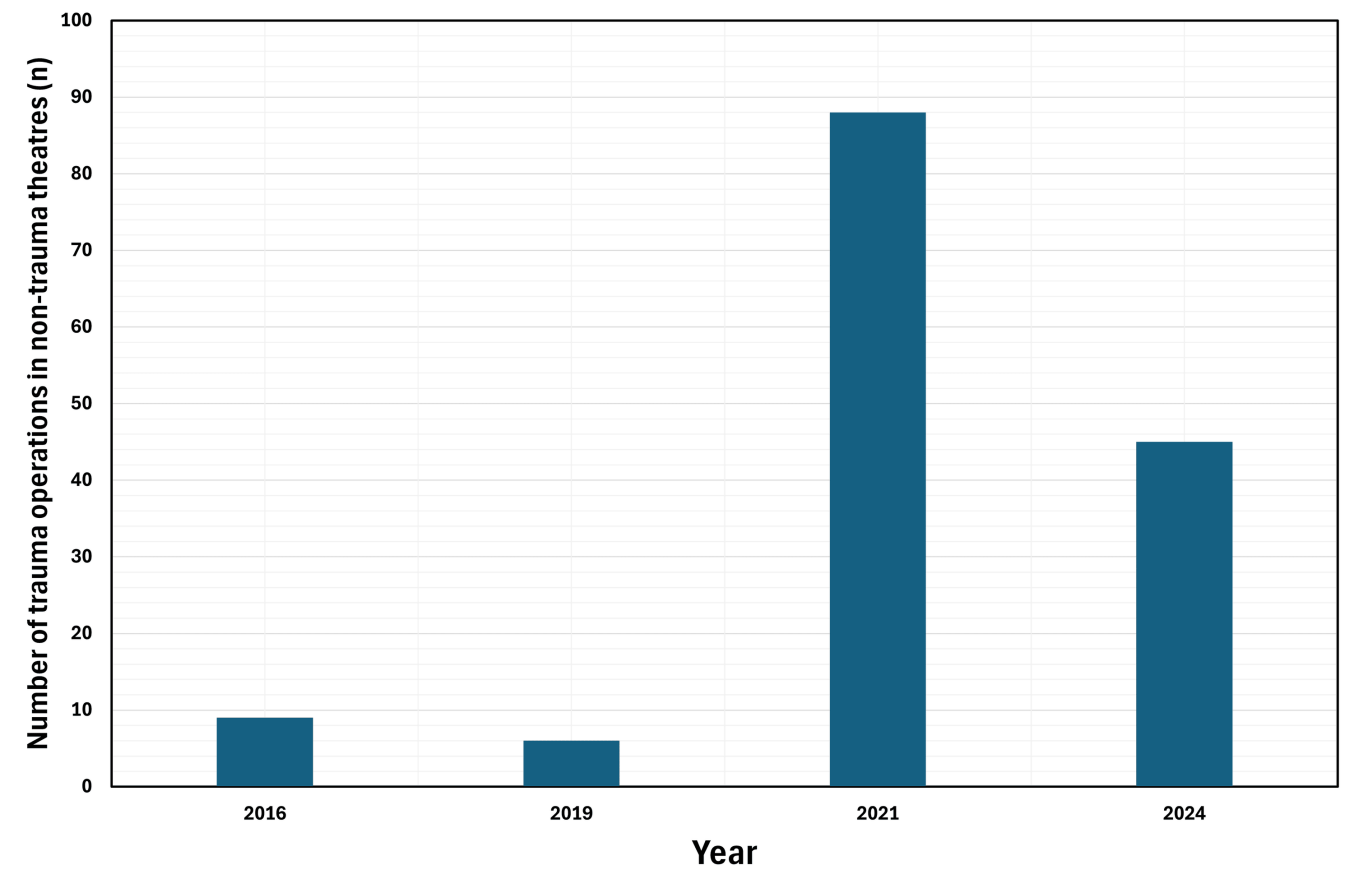
Number of trauma operations being performed on elective lists or ad hoc additional trauma lists by year Bar chart demonstrating the increasing number (n) of trauma operations being peformed outside of trauma theatres in elective lists or ad hoc additional trauma lists.

The number of trauma operations performed in elective or ad hoc additional trauma lists opened to cope with trauma burden has increased considerably, from nine and six in 2016 and 2019 respectively, to 88 in 2021 and 45 in 2024 (Figure 5).

## Discussion

As evidenced, this major trauma centre has seen a significant overall upward trend in common trauma operations. There has been a substantial increase in many time-sensitive operations, including open fractures, femoral shaft fractures and hip fractures, despite no permanent increase in dedicated trauma lists. This is in keeping with findings by Shah, Judge and Griffin [14], who found the incidence of open fractures had increased from 2.50 per 100,000 people in 2008 to 7.03 per 100,000 people in 2019, as well as Jennison and Brinsden [15], who found an upward trend in fracture related admissions in England between 2004/2005 and 2013/2014 including hip, femoral shaft and many upper limb fractures.

This rise corresponds to an increase in the number of patients discussed in the daily trauma meeting. However, the number of trauma operations being performed has not drastically changed. To explain why there has been no significant increase in operative output on trauma lists despite a rise in trauma, we note that the service has made several adaptations.

Firstly, the recent expansion of trauma consultants’ semi-elective lists has allowed for many operation types to be migrated out of the trauma theatres. These lists are predominantly reserved for follow-up trauma patients requiring further procedures, including but not limited to revisions, removal of metalworks and operative management of non-unions and avascular necrosis, many of which used to be routinely performed in trauma theatres. These lists also regularly house overflow trauma, especially ambulatory trauma, when capacity allows. This is in keeping with recommendations from the Getting It Right First Time (GIRFT) initiative [16], which suggested expansion of semi-elective day-case trauma lists to reduce burden on orthopaedic inpatient beds and trauma theatres.

Secondly, the restructuring and improvements to the region’s PJI service has resulted in fewer PJI operations being performed in trauma theatres, other than acutely unwell patients admitted via the emergency department or straight from clinic. These patients are now often referred directly to the PJI team, where they are routinely managed on PJI specialists’ dedicated lists.

Finally, there has been a large rise in the number of trauma operations performed outside of trauma theatres on elective lists or ad hoc additional lists opened to manage the trauma burden. This was especially the case in 2021, where 88 operations were performed outside of trauma theatres. This high number can be partially explained by the increased capacity for opening ad hoc trauma lists in the immediate post-pandemic period prior to elective services returning to full capacity, and partially by it being before the increase in semi-elective trauma lists which today house many of these operations.

The UK population is projected to continue to rise and age [17]. From this, we can infer that the volume of trauma is projected to increase with it. Policymakers and NHS Trusts need to factor in these projections into trauma service planning in the coming decade and beyond. If we fail to account for these rises, demand for safe and timely trauma services will quickly exceed capacity. Patients would likely see delays in receiving operations. In the case of patients with hip fractures, this might mean longer periods of immobilisation associated with higher morbidity and mortality [18], and Best Practice Tariff targets being more regularly missed with a subsequent loss of funding. Delays to surgery might also result in longer periods of time off work, with knock on effects on the economy.

To adapt to the rise in caseload, trauma services will need to expand their operative capacity. This may take the form of expanding semi-elective ambulatory trauma lists, as recommended by the GIRFT specialty report on adult orthopaedic trauma [16]. Semi-elective ambulatory lists have the advantage of being less prone to last-minute changes to accommodate for urgent cases, allowing for better preparation, fewer delays and fewer cancellations. Permanent additional theatre sessions in trauma theatres would be necessary if time-sensitive trauma continues to regularly exceed trauma theatre capacity.

Service planning would need to be tailored to the hospital and region’s specific set of circumstances. Certain trauma networks might experience higher volumes of violent crimes whilst others might receive more road traffic accidents or have a higher proportion of low-energy trauma in older persons, and the services would have to be tailored accordingly. Other networks may already have greater theatre capacity but lack the workforce size or experience to fully utilise it. Policymakers will have to work closely with local actors to ensure service planning accounts for these discrepancies between regions.

The limitations of this study include relatively small sample sizes yielding limited statistically significant results. It being a single-centre study was useful for exploring the specific adaptations this trauma service has made but limits the generalisability of the results. Trauma workload is notorious for its ebb and flow, and the study design being limited to specific months might not capture this variation sufficiently.

Suggestions for further research include exploring whether the rise in trauma seen to date has had a detrimental impact on time to surgery. This study has focussed on orthopaedic trauma, but service planning would need to factor in trauma in other specialties. Further epidemiological data exploring cause of injury might help target funding for preventative strategies, for example improving road safety awareness, suicide prevention initiatives or reducing violent crime. Whilst the use of local data allowed for insight into theatre utilisation and service adaptations, future studies could make use of the National Major Trauma Registry to more accurately plot and predict trends in trauma prevalence and incidence to aid service planning.

## Conclusions

Orthopaedic trauma services in the UK are experiencing substantial increases in workload, as the volume of trauma rises with the growing and ageing population. This MTC has seen a significant rise in many common fracture operations and adapted to this rise by expanding its semi-elective trauma service, increasing the availability of ad hoc trauma lists, introducing improved referral pathways, and migrating certain operation types from trauma theatres to elective theatres. Service planners need to account for the rising volume of trauma to prevent trauma services from becoming overwhelmed, which would lead to delays for patients receiving safe and timely treatment. Trauma services might increase capacity through the expansion of semi-elective ambulatory trauma lists and more dedicated trauma theatre sessions.

## References

[1] (2025). Office for National Statistics (ONS): Population estimates for the UK, England, Wales, Scotland and Northern Ireland: mid-2024. https://www.ons.gov.uk/peoplepopulationandcommunity/populationandmigration/populationestimates/bulletins/annualmidyearpopulationestimates/mid2024.

[2] (2025). Office for National Statisics (ONS): Health state life expectancies in England, Northern Ireland and Wales: between 2011 to 2013 and 2020 to 2022. Wales: between.

[3] Kingston A, Robinson L, Booth H, Knapp M, Jagger C (2018). Projections of multi-morbidity in the older population in England to 2035: estimates from the Population Ageing and Care Simulation (PACSim) model. Age Ageing.

[4] Willers C, Norton N, Harvey NC (2022). Osteoporosis in Europe: a compendium of country-specific reports. Arch Osteoporos.

[5] Capone A, Congia S, Civinini R, Marongiu G (2017). Periprosthetic fractures: epidemiology and current treatment. Clin Cases Miner Bone Metab.

[6] (2025). Gov.UK: Plan for change: Build an NHS fit for the future. https://www.gov.uk/missions/nhs.

[7] (2025). National Audit Office: NHS England’s management of elective care transformation programmes. https://www.nao.org.uk/wp-content/uploads/2025/03/nhs-englands-management-of-elective-care-transformation-programme.pdf.

[8] Poutoglidou F, Elliot R (2025). Trauma in the shadows: the unseen consequences of elective surgery pressures. Bull R Coll Surg Engl.

[9] (2025). National Hip Fracture Database: Room for improvement: hip fracture care in 2024. https://www.nhfd.co.uk/reportopen/NHFD+2025+Annual+Report.

[10] Hull PD, Johnson SC, Stephen DJ, Kreder HJ, Jenkinson RJ (2014). Delayed debridement of severe open fractures is associated with a higher rate of deep infection. Bone Joint J.

[11] Obey MR, Clever DC, Bechtold DA (2022). In-hospital morbidity and mortality with delays in femoral shaft fracture fixation. J Orthop Trauma.

[12] Lankester BJ, Paterson MP, Capon G, Belcher J (2000). Delays in orthopaedic trauma treatment: setting standards for the time interval between admission and operation. Ann R Coll Surg Engl.

[13] Park C, Sugand K, Nathwani D, Bhattacharya R, Sarraf KM (2020). Impact of the COVID-19 pandemic on orthopedic trauma workload in a London level 1 trauma center: the "golden month". Acta Orthop.

[14] Shah A, Judge A, Griffin XL (2022). Incidence and quality of care for open fractures in England between 2008 and 2019 : a cohort study using data collected by the Trauma Audit and Research Network. Bone Joint J.

[15] Jennison T, Brinsden M (2019). Fracture admission trends in England over a ten-year period. Ann R Coll Surg Engl.

[16] Getting It Right First Time (2025). Getting it right first time. Adult orthopaedic trauma: GIRFT programme national specialty report. https://gettingitrightfirsttime.co.uk/wp-content/uploads/2025/09/Adult-Orthopaedic-Trauma-Nov24L-exec-summary.pdf.

[17] (2025). Office for National Statistics (ONS): National population projections: 2022-based. https://www.ons.gov.uk/peoplepopulationandcommunity/populationandmigration/populationprojections/bulletins/nationalpopulationprojections/2022based.

[18] Simunovic N, Devereaux PJ, Bhandari M (2011). Surgery for hip fractures: does surgical delay affect outcomes?. Indian J Orthop.

